# The impact of microbially modified metabolites associated with obesity and bariatric surgery on antitumor immunity

**DOI:** 10.3389/fimmu.2023.1156471

**Published:** 2023-05-16

**Authors:** Meng Wang, Yuhong Huang, Meiling Xin, Tianxing Li, Xueke Wang, Yini Fang, Shufei Liang, Tianqi Cai, Xiaoxue Xu, Ling Dong, Chao Wang, Zhengbao Xu, Xinhua Song, Jingda Li, Yanfei Zheng, Wenlong Sun, Lingru Li

**Affiliations:** ^1^ School of Life Sciences and Medicine, Shandong University of Technology, Zibo, Shandong, China; ^2^ National Institute of Traditional Chinese Medicine Constitution and Preventive Medicine, Beijing University of Chinese Medicine, Beijing, China; ^3^ College of Life Science, Yangtze University, Jingzhou, Hubei, China; ^4^ Institute of Basic Theory for Chinese Medicine, China Academy of Chinese Medical Sciences, Beijing, China; ^5^ The Second Clinical Medical College, Henan University of Chinese Medicine, Zhengzhou, Henan, China; ^6^ Basic Medical College, Zhejiang Chinese Medical University, Hangzhou, Zhejiang, China

**Keywords:** obesity, bariatric surgery, cancer, metabolites, anti-tumor immunity

## Abstract

Obesity is strongly associated with the occurrence and development of many types of cancers. Patients with obesity and cancer present with features of a disordered gut microbiota and metabolism, which may inhibit the physiological immune response to tumors and possibly damage immune cells in the tumor microenvironment. In recent years, bariatric surgery has become increasingly common and is recognized as an effective strategy for long-term weight loss; furthermore, bariatric surgery can induce favorable changes in the gut microbiota. Some studies have found that microbial metabolites, such as short-chain fatty acids (SCFAs), inosine bile acids and spermidine, play an important role in anticancer immunity. In this review, we describe the changes in microbial metabolites initiated by bariatric surgery and discuss the effects of these metabolites on anticancer immunity. This review attempts to clarify the relationship between alterations in microbial metabolites due to bariatric surgery and the effectiveness of cancer treatment. Furthermore, this review seeks to provide strategies for the development of microbial metabolites mimicking the benefits of bariatric surgery with the aim of improving therapeutic outcomes in cancer patients who have not received bariatric surgery.

## 1 Introduction

Cancer is one of the major diseases threatening human life and health. According to GLOBOCAN 2020, there will be approximately 19.3 million new cancer cases and at least 10 million cancer deaths worldwide in 2020 (1). It was predicted that by 2022, there would be approximately 4.82 million new cancer cases and at least 3.21 million cancer deaths in China and approximately 2.37 million new cancer cases and at least 0.64 million cancer deaths in the United States (2). In many countries, cancer is increasingly becoming the main cause of death. At present, frequently used treatment methods that target tumors include radiotherapy, chemotherapy, surgery, and targeted therapy. In December 2013, Science magazine named tumor immunotherapy one of the top 10 technological breakthroughs of the year (3). American scientist James P. Allison and Japanese scientist Tasuku Honjo were awarded the 2018 Nobel Prize in Physiology or Medicine for their discovery that negative immune regulation can be used to treat cancer. The current clinical applications of tumor immunotherapy include immune checkpoint inhibitor therapy, adoptive cellular immunotherapy, and cancer vaccines, among others (4). Clinically, tumor immunotherapy has shown clear efficacy in patients with different cancer diagnoses, including long-term remission after treatment (5). Moreover, tumor immunotherapy has the potential to cure a small number of patients who develop metastases and were previously thought to be incurable (6, 7). Tumor immunotherapy is a novel treatment approach that has rapidly developed in recent years (8). Unlike traditional therapy, tumor immunotherapy primarily targets the immune system rather than tumor cells. The immune system prevents cancer progression because of its ability to recognize and destroy abnormal cells. Tumor immunotherapy can eliminate tumor cells by strengthening the body’s innate immune defense and reshaping the immune microenvironment (9). Therefore, reactivating the body’s immune system is conducive to killing tumor cells.

Although immunotherapy has benefited many patients, these patients remain a minority (10). Two studies found that the gut microbiome had a significant impact on cancer immunotherapy in mouse models (11, 12). Furthermore, scientists have observed that the impact of the gut microbiome on cancer immunotherapy is variable in cancer patients; specifically, some gut microbiome metabolites can significantly strengthen cancer immunotherapy and, thus, are considered favorable. There is no consensus on what is considered favorable. Researchers from France found that *Akkermansia muciniphila* was beneficial for immunotherapy against epithelial tumors (13). Researchers from North America discovered that the beneficial gut microbes included *Collinsella aerofaciens*, *Enterococcus faecium*, and *Bifidobacterium longum* in patients with metastatic melanoma (14), while researchers from South America identified Ruminococcaceae as part of a favorable gut microbiome in patients with melanoma (15). Furthermore, researchers from China identified *Bifidobacterium longum*, *Prevotella copri*, and *Alistipes putredinis* as beneficial gut microbes for patients with non-small cell lung cancer (NSCLC) receiving immunotherapy (16). These results imply that regional differences influence what is considered a favorable microbiome. Therefore, scientists have begun to explore the relationships between microbial metabolites and enhanced cancer immunotherapy.

Obesity is defined as body mass index (BMI) ≥ 30 kg/m^2^ (weight in kilograms divided by height in meters squared) and is the most important risk factor for human health. Unfortunately, the long-term effectiveness of dietary intervention is limited. No effective drugs are currently available for individuals who have morbid obesity. Therefore, bariatric surgery is widely applied and is a high-efficiency treatment option for most patients with obesity. Bariatric surgery is recommended for obese patients with a BMI > 35 to 40 kg/m^2^ and other comorbidities. Generally, bariatric surgery has become an option for inducing weight loss, and numerous studies have recently shown that it also alters the gut microbiota profile. Indeed, many researchers have reported that alterations in microbial metabolites after bariatric surgery are beneficial in the context of other diseases, such as type 2 diabetes (17–20). However, the impact of the microbial metabolites associated with bariatric surgery on cancer and antitumor immunity requires further investigation.

In this review, we introduce and analyze the changes in microbial metabolites caused by bariatric surgery and the effects of the subsequent gut-derived metabolites on anticancer immunity. Furthermore, we attempt to clarify the relationship between the changes in microbial metabolites caused by bariatric surgery and the effectiveness of cancer treatment with the aim of providing strategies for cancer patients with obesity.

## 2 Bariatric surgery

The World Health Organization has listed obesity as one of the five major diseases that seriously endanger human health. It not only affects quality of life but is also involved in several complications, such as cardiovascular and cerebrovascular diseases, diabetes, and fatty liver. In recent years, several weight loss methods and drugs have emerged, but most have been abandoned due to associated adverse reactions, lack of efficacy, and relapse; however, surgical treatment is currently recognized as an effective and safe method to treat morbid obesity.

The National Institutes of Health established guidelines for bariatric surgery in 1991. Surgical weight loss refers to the use of medical and surgical means to improve the overall symptoms of patients with obesity. For patients with severe obesity, those with early complications such as diabetes, those who are unwilling to change their lifestyle, or those who cannot effectively control their weight after lifestyle adjustments, surgical treatment has remained the most effective method. During the 21st century, the six most popular bariatric surgical methods for weight reduction have been laparoscopic sleeve gastrectomy, laparoscopic gastric bypass surgery, laparoscopic gastric band surgery, gastric balloon surgery, single-anastomosis duodenoileal bypass with sleeve gastrectomy surgery and stomach intestinal pylorus sparing surgery. Bariatric surgery is a long-term modulator of the gut microbiota that influences not only its composition but also its function, as the efficiency of energy extraction from the diet is lowered, which is a potential mechanism that may be associated with the sustained beneficial metabolic effects observed in successful interventions (21).

### 2.1 Laparoscopic sleeve gastrectomy

Gastrectomy, also known as laparoscopic sleeve gastrectomy or vertical sleeve gastrectomy, was first proposed by Hess and Marceau in 1988 and is often used as the first procedure in a laparoscopic Roux-en-Y gastric bypass (RYGB) procedure. It was not used as a standalone procedure until Johnston et al. in 1993 (22). The principle of laparoscopic sleeve gastrectomy involves the use of vertical laparoscopic removal of the larger curvature of the stomach to form a 150 cc small stomach that can accommodate approximately 4 to 5 ounces of food. Without altering the physiological state of the gastrointestinal tract or interfering with the normal digestion and absorption process of food, approximately two-thirds of the stomach is removed in patients with obesity, directly reducing stomach volume, limiting food intake, and leading to less hunger in patients with obesity. Since the feasibility of this technique is high, it has received attention from an increasing number of specialists, patients, and medical service institutions and has been widely used in clinical practice (23). Sleeve gastrectomy is also viewed favorably since there is no need to introduce foreign substances into the body, and this surgical technique results in significant weight loss. Specifically, sleeve gastrectomy could help patients with obesity lose more than 20% of their total body weight within one year of surgery, with a low occurrence of weight regain. Moreover, sleeve gastrectomy has a positive therapeutic effect in individuals with type 2 diabetes; therefore, it is widely used in Europe to treat obesity and diabetes. However, a number of complications may also occur after sleeve gastrectomy, such as acid reflux, sleeve narrowing, excessive weight loss, vitamin deficiency, and weight regain (24). Therefore, more meaningful studies are needed to determine the value of sleeve gastrectomy surgery in the bariatric process.

### 2.2 Laparoscopic gastric bypass surgery

Laparoscopic RYGB is a complex operation that alters the structure of the intestine, eliminating much of the stomach, which reduces the capacity of the stomach and the length of the small intestine. This irreversible surgery was first reported by Wesley and Alan in 1994 and is the most frequent type of bariatric surgery in the U.S (22). During gastric bypass surgery, a small gastric pouch is formed by dividing the stomach and transecting the jejunum. The distal portion of the jejunum is attached to the gastric pouch to form the Roux limb, and the proximal portion is further reattached to the Roux limb to form the biliopancreatic limb (25). The operation divides the stomach of the patient into an upper and lower region, with only ^1^/_6_ to ^1^/_10_ of the original stomach being used to store food. Next, a “fork road” is opened at the incision of the small stomach to connect to a section of the small intestine, which rearranges the position of the small intestine, changing the path of food through the digestive tract and resulting in a slower rate of gastric emptying and reduced absorption due to the shortened length of the small intestine, thereby promoting weight loss in the patient.

Among surgeries targeting obesity and diabetes, gastric bypass is a quick and frequently used surgery and the most effective treatment for patients with obesity and type 2 diabetes. However, laparoscopic gastric bypass surgery is only suitable for patients with BMI ≥ 45 kg/m² (extreme obesity) or who have BMI ≥ 35 kg/m² plus other medical conditions related to obesity, such as diabetes or heart disease (26). Moreover, several complications are associated with abdominal surgery, including infection, massive hemorrhage, hernia, intestinal obstruction and venous thromboembolism. Therefore, a physician carefully reviews the health status of a patient before commencing surgery.

### 2.3 Adjustable gastric band surgery

The adjustable gastric band is a special silicone band containing an adjustable water sac. The gastric band is tied at the upper end of the stomach, and a “small stomach sac” similar to that produced by a gastric septum is generated above the stomach. The volume of the small stomach sac is approximately 20~330 mL. Adjustable gastric band surgery is one of the best treatment surgeries for obesity and is the most widely used form of bariatric surgery in the United States and Europe. However, there are certain prerequisites that must be met before performing the operation, including the following: (1) the patient’s age is between 18 and 65 years old; (2) patients must have severe obesity with BMI>32 and have no other complications; (3) surgery is only suitable for patients who have not responded to medical treatment and other weight loss strategies. Under normal circumstances, laparoscopic adjustable gastric banding can change the eating habits of patients with obesity, resulting in sustained weight loss. Advantages include the following: (1) The procedure has a low mortality rate (1 in 2000 patients compared with 2% for RYGB within 30 days of each procedure); (2) The procedure does not require the removal or destruction of the stomach organs and is completely reversible; (3) The process is associated with short hospital stays and quick recovery; (4) No additional operation is required to adjust the straps; and (5) This procedure has less serious complications than gastric bypass surgery. However, the failure rate of this surgery is very high, with the main reasons for failure including slippage of the adjustable gastric band, infection, and intolerance (22–24). These late complications are usually corrected by surgery. More research is needed to assess the pros and cons of gastric banding surgery.

### 2.4 Gastric balloon surgery

The intragastric balloon procedure was developed in 1985 with the Garren-Edwards Bubble as a minimally invasive temporary weight loss treatment. Intragastric balloon surgery, also known as gastric water balloon bariatric surgery, is very useful for weight loss. It works through the insertion of a silicon water balloon into the stomach using a gastroscope and the injection of saline into the balloon to occupy the space in the stomach, induce a feeling of fullness and help control appetite (27). Gastric water balloons first began to be used in Europe in the 1990s. The use of intragastric balloons for the prevention of obesity was approved by the US Food and Drug Administration in 2015 (27). In addition, gastric water balloons are correlated with significantly higher weight loss in Asian populations than in European populations. Obesity in Asian populations is thought to be related to a family history of dysregulated blood glucose control. Gastric water balloons can provide the patient with a feeling of fullness while regulating intestinal anti-insulin secretion. In addition to the effect of weight loss, it has significantly improved diabetes in Asian diabetic patients.

Gastric balloon surgery is beneficial because it (1) is relatively risk-free; (2) has few side effects; (3) is reversible; and (4) is suitable for adolescents. Furthermore, gastric water balloons have been shown to be effective in patients with bulimia, but the efficacy is still being assessed. However, the evidence is insufficient due to the small number of people who have undergone gastric balloon surgery and the lack of long-term follow-up. Therefore, more studies are necessary to confirm the beneficial effect of gastric balloon surgery on weight loss.

### 2.5 Single-anastomosis duodenoileal bypass with sleeve gastrectomy surgery

Single-anastomosis duodenoileal bypass with sleeve gastrectomy surgery (SADI-S), a novel bariatric surgery, combines the characteristics of sleeve gastrectomy surgery and jejunoileal bypass surgery. The first step in SADI-S surgery is sleeve gastrectomy, which is performed over a wide 54 French gastric bougie. This step directly limits the intake of food. Subsequently, duodenoileal diversion is performed 250-300 cm from the ileocecum, eliminating the digestive limb while expanding the common limb of the biliopancreatic diversion (28). The procedure has similar features to laparoscopic RYGB surgery in terms of enhanced metabolic improvement and durability. The procedure can limit caloric intake by reducing gastric volume and reduce the absorption of ingested nutrients through the removal of part of the small intestine (29). Sánchez-Pernaute et al. first used SADI-S for the treatment of obesity in 2007 and reported the safety and good weight loss effects of the surgery in 2009 (30, 31). In 2020, the American Society for Metabolic and Bariatric Surgery stated that SADI-S is an appropriate bariatric surgery (32). Similarly, in 2021, the International Federation of Obesity and Metabolic Disorders Surgery declared that SADI-S will help people who are obese lose a significant amount of weight and maintain the weight loss over time while improving their metabolic health (33). In addition, SADI-S reduces the incidence of obesity-related complications (type 2 diabetes, dyslipidemia, hypertension), and the surgery has very few side effects (34, 35). SADI-S is a promising bariatric surgery.

### 2.6 Stomach intestinal pylorus sparing surgery

The single anastomosis duodenal switch or stomach intestinal pylorus sparing surgery (SIPS) is a modified duodenal switcher that combines vertical sleeve gastrectomy surgery with duodenal-enteral anastomosis surgery (36). The features of SIPS are the preservation of longer bowel length and the ileocecal valve, which effectively reduce short bowel syndrome. Additionally, preservation of the pylorus helps to maintain the normal physiological functions of the body, such as regulating the rate of solid food emptying from the stomach and reducing the risk of dumping syndrome (37). Cottam et al. showed that SIPS was associated with fewer short- and long-term complications and a higher rate of regression of type 2 diabetes than RYGB surgery (38). However, as SIPS is a relatively new procedure, follow-up studies on the surgery are inadequate, and further studies are still needed to refine the knowledge regarding this surgery.

## 3 Bariatric surgery and gut microbiota metabolites

**Figure 1:**
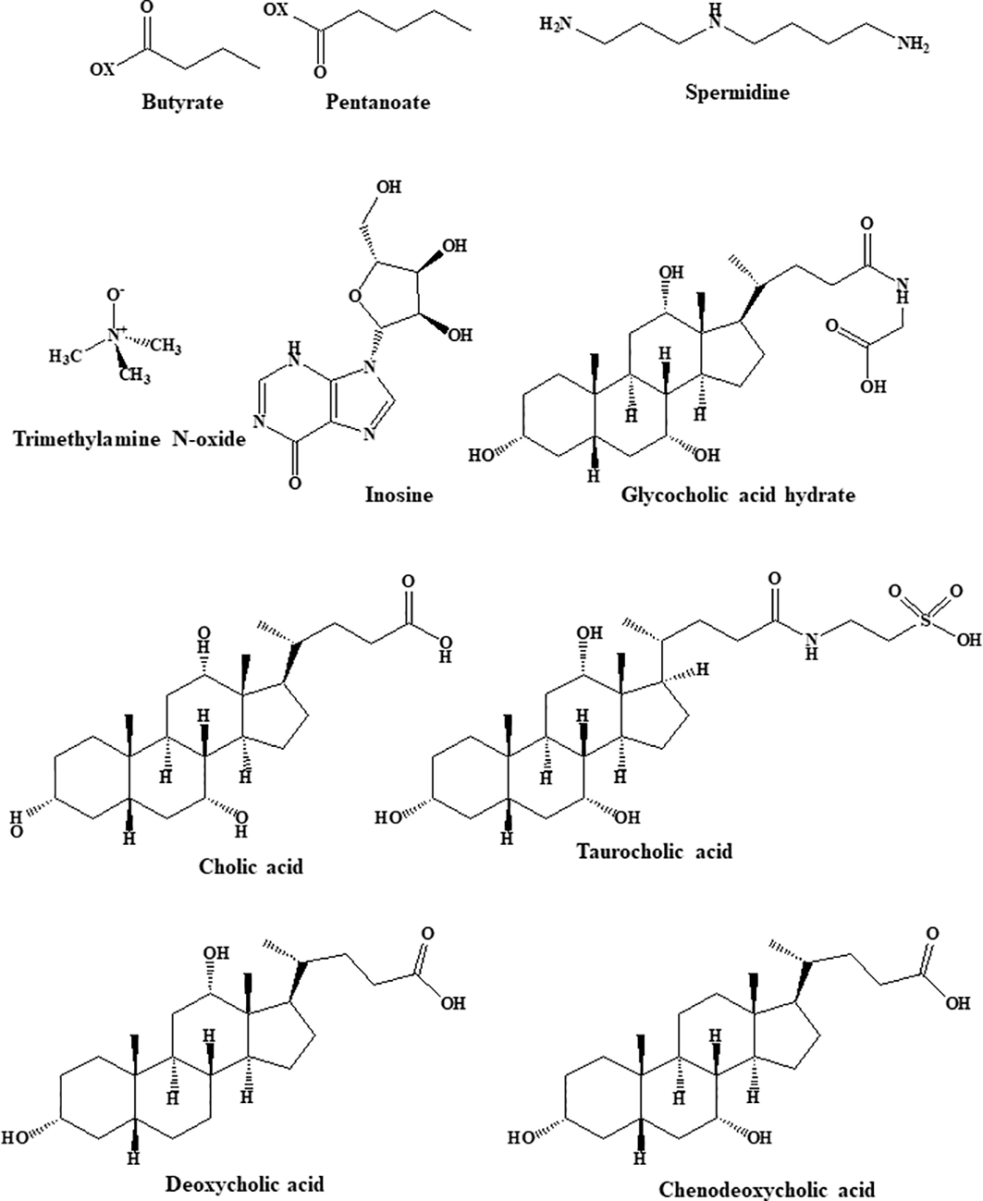
Structural formula of gut microbiota metabolites.

Multiple studies have indicated that alterations in the gut microbiota and its metabolites are strongly associated with the occurrence of obesity. For example, obesity is connected to an increase in the ratio of *Firmicutes* to *Bacteroidetes* abundance and is associated with a dysbiotic energy-harvesting microbiome. During digestion, the gut microbiota produces large quantities of physiologically active substances, including metabolically beneficial short- and long-chain fatty acids (SCFAs and LCFAs), bile acids, and tryptophan metabolites (39). The structures of the gut microbiota metabolites are shown in **Figure 1**.

The function of gut microbiota metabolites in obesity recovery is also very interesting. SCFAs (especially butyrate, propionate and acetate) are the main class of gut-derived beneficial metabolites and are produced when the microbiota metabolizes complex dietary carbohydrates (69). Foods positively associated with the production of SCFAs include sugar, red/orange vegetables, and milk, whereas animal protein is negatively correlated with SCFA production (41). It has been well documented that SCFAs are strongly associated with metabolic syndromes, particularly type 2 diabetes, hypertension and obesity (70, 71). Many studies have demonstrated that SCFAs play a key role in energy metabolism and obesity by inhibiting appetite, suppressing lipogenesis and inducing the browning of white adipose tissues (72). Specifically, SCFAs exert appetite suppressant effects by directly interacting with individual neurons isolated from nodose ganglia to induce intracellular Ca^2+^ signaling and by increasing serum levels of peptide YY (PYY), glucagon-like peptide 1 (GLP-1), and leptin (73, 74). Additionally, SCFAs reduce appetite and suppress fat accumulation by regulating related genes (acetyl-CoA carboxylase-1, trifunctional protein alpha and carnitine palmitoyl transferase II) and hormones (such as tumor necrosis factor receptor superfamily member 9, mitochondrial transcription factor A, free fatty acid receptor 2, and cytochrome-C oxidase IV, 75–77). Other studies have shown that SCFAs inhibit lipid synthesis and prevent obesity-related metabolic disorders by reducing the levels of hepatic triglycerides and increasing the yield of odd-chain fatty acids (78). Finally, SCFAs can increase energy expenditure and weight loss by inhibiting white adipose tissue lipolysis and inducing white adipose tissue browning (79).

Studies have shown that high levels of trimethylamine N-oxide (TMAO) are associated with the risk of serious cardiovascular events, such as heart attacks and strokes. Since cardiovascular disease and obesity are closely linked, researchers hypothesized that TMAO might also be involved in obesity-related metabolic pathways. Some recent studies suggest that increased concentrations of TMAO increase the risk of obesity (80). Flavin-containing monooxygenase 3 (FMO3) can convert TMAO to its active form. A study revealed that the deletion or inactivation of the FMO3 gene in mice was able to prevent obesity even if the mice were fed a high-fat, high-calorie diet (80). Interestingly, the level of TMAO in type 2 diabetic patients was found to be more than twofold higher after bariatric surgery (47). Therefore, an increase in TMAO levels will be detrimental to weight loss.

Primary bile acids are synthesized from cholesterol in the liver and then transformed into secondary bile acids by bacteria in the gut. Bile acids not only promote the absorption of meals, fats, and fat-soluble molecules but also act as signaling molecules, which activate the G-protein-coupled receptor (TGR5) and the nuclear receptor (FXRα, 81). Bile acids can improve metabolism and play an anti-obesity role by activating TGR5 and FXR in peripheral tissues (82). In addition, Watanabe et al. found that bile acids can prevent insulin resistance and obesity in mice by increasing energy depletion in brown adipose tissue (83, 84).

Serum polyamines are one of the important metabolites derived from amino acid metabolism and are closely related to the metabolic status of patients with obesity (52). The dysregulation of polyamine metabolism affects the regulation of glucose, lipids, and energy homeostasis. Many studies have shown that polyamines (spermidine and spermine) can act as reactive oxygen species (ROS) scavengers to protect DNA from oxidative damage (85). Spermidine is found in some foods, such as fruits, nuts, wheat germ, soybeans, and vegetables. It is protective against cancer, heart disease, metabolic diseases, and neurodegeneration (86). Spermine, spermidine and their precursor diamine putrescine are the major polyamines in mammalian cells (87). Spermidine-mediated protection involves the regulation of inflammatory responses, lipid metabolism, intestinal barrier function and thermogenesis (88). Spermidine plays a vital role in enhancing metabolism and weight loss through mechanisms such as alteration of gene expression, enhancement of energy metabolism, glucose and lipid metabolism, and autophagy. Specifically, spermidine activates the AMPK pathway to reduce adipogenic gene expression, increases autophagy in white adipose tissue, modulates the activity of neurological disease targets (including AMPA, NMDA, CB1, GABA, various astrocyte junction channels and components of the neuroinflammatory cascade), reduces metabolic endotoxemia and improves intestinal barrier function (89).

Inosine is a signaling molecule in cells and broadly impacts the genome, transcriptome and proteome (90). Recent research published in Nature shows that inosine released from apoptotic brown adipocytes activates adenosine-2A and -2B receptors (A2A/A2B), which stimulate thermogenesis through the cAMP-p38 axis. It is possible that the browning of adipose tissue and increased energy expenditure may be effective in preventing obesity (91). The study by Kong et al. similarly established that inosine promotes adipocyte browning and improves diet-induced obesity (92). Inosine can be a potential weight loss drug due to its specific thermogenic effect. The role of bariatric surgery in the production of gut microbiota metabolites is of great interest. And the representative changes are shown in **Table 1**.

**Table 1:** Changes in intestinal flora metabolites after bariatric surgery.

Bariatric surgery	Representative gut microbiota and their metabolic characteristics					Gut microbiota metabolite	Variation tendency	References
	Phylum	Genus	Species	Trend	Reference			
Laparoscopic Sleeve Gastrectomy	*Firmicutes*	*Bulleidia*		↑	(40)	Butyrate		
		*Roseburia*	*intestinalis*				↓	(41–43)
		*Faecalibacterium*	*prausnitzii*				↑	(44, 45)
		*Coprococcus*	*comes*	↓				
		*Streptococcus*	*parasanguinis*	↑	(42)	Pentanoate	↓	(41, 44, 46)
		*Anaerotruncus*	*hadrus*					
		*Lachnoclostridium*				Trimethylamine N-oxid	——	(46–48)
	*Bacteroidetes*	*Odoribacter*	*splanchnicus*					
		*Alistipes*	*finegoldii*					
			*putredinis*			Bile acids	↑	(49–51)
	*Proteobacteria*	*Klebsiella*						
	*Actinobacteria*	*Actinomyces*				Spermidine	↑	(52)
		*Bifidobacterium*						
	*Verrucomicrobia*	*Akkermansia*	*muciniphila*					
	*Bacteroidetes*	*Bacteroides*	*finegoldii*	↓				
Laparoscopic Gastric Bypass Surgery			*stercoris*			Butyrate	↓	(25, 41, 42)
			*ovatus*				↑	(53, 54)
	*Firmicutes*	*Dorea*	*longicatena*					
			*formicigenerans*			Pentanoate	↓	(41, 42, 46)
		*Fusicatenibacter*	*saccharivorans*				↑	(55)
		*Roseburia*						
		*Eubacterium*	*rectale*			Inosine	↓	(56)
			*hallii*				↑	(57)
	*Firmicutes*	*Roseburia*	*intestinalis*	↑	(40)			
		*Lactobacillus*		↓		Trimethylamine N-oxid	↑	(47, 56, 58, 59)
		*Faecalibacterium*	*prausnitzii*					
		*Coprococcus*	*comes*					
	*Actinobacteria*	*Bifidiobacterium*				Bile acids	↑	(46, 60–64)
	*Proteobacteria*	*Klebsiella*		↑				
		*Escherichia*						
		*Escherichia*	*coli*					
	*Verrucomicrobia*	*Akkermansia*		↑				
	*Bacteroidetes*	*Bacteroide*		↓	(65)			
	*Firmicutes*	*Blautia*						
Adjustable Gastric Band Surgery					(40)	Butyrate	↓	(42)
	*Proteobacteria*	*Escherichia*	*coli*	↑		Pentanoate	↓	(42)
	*Firmicutes*	*Roseburia*	*intestinalis*	↓		Bile acids	——	(63)
							↑	(66, 67)
							↓	(60)
Gastric balloon surgery	*——*	*——*	*——*	*——*	*——*	*——*	*——*	——
Single-anastomosis Duodeno-ileal Bypass with Sleeve Gastrectomy Surgery	*Proteobacteria*	*Enterobacteriaceae*		↑	(55)			
		*Sutterellaceae*	*Sutterella*					
	*Firmicutes*	*Clostridiaceae*	*Clostridium*			Butyrate	↑	(55)
		*Oscillospira*		↓				
		*Ruminococcus*						
		*Oscillospiraceae*	*Oscillibacter*			Bile acids	↑	(68)
		*Christensenellaceae*						
Stomach Intestinal Pylorus Sparing Surgery	——	——	——	——	——	——	——	——

“↑” represents an increase in metabolite concentration; “↓” represents a decrease in metabolite concentration.

### 3.1 Laparoscopic sleeve gastrectomy and gut microbiota metabolites

#### 3.1.1 Laparoscopic sleeve gastrectomy and SCFAs

Butyrate is a four-carbon SCFA that plays a vital role in regulating the impact of the gut microbiota on gut health, obesity and local and systemic immunity (93, 94). However, the levels of SCFAs in patients after metabolic surgery remain controversial. The Clostridium cluster of the phylum Firmicutes mainly produces butyrate and includes bacteria such as *Anaerostipes*, *Roseburia*, *Faecalibacterium*, *Coprococcus*, *Subdoligranulum*, *Anaerobutyricum*, and *Eubacterium* (95). In a study by Davies et al., the relative abundance of Firmicutes decreased after sleeve gastrectomy (40). Meijer et al. observed that gastrectomy resulted in a significant reduction in the levels of straight-chain SCFAs by reducing the abundance of primary microbial degraders (responsible for producing acetate) and secondary degraders (which produce butyrate, valerate, and propionate, 41). Yu et al. demonstrated that beta diversity and fecal metabolites were also altered postgastrectomy and observed a reduction in the levels of butyrate, indoles, and caffeine metabolites (42). However, Nugent et al. demonstrated a significant upward trend in the levels of butyrate-producing bacteria, such as *E. rectale, Roseburia* spp., and *F. prausnitzii*, within 1 month postgastrectomy, which was accompanied by a relative increase in serum butyrate levels (44). Similarly, a study by Guo et al. revealed indications of improved metabolism and an increased number of SCFA-producing bacteria, in addition to increased butyrate levels in obese diabetic mice after gastrectomy (45). Farup and Valeur studied alterations in SCFAs after bariatric surgery and observed reductions in the levels of straight-chain SCFAs (butyrate, acetate, and propionate) and increases in the levels of branched-chain SCFAs (isocaproatee, isovalerate, and isobutyrat, 43).

Pentanoate is an inexpensive and effective immunomodulator with the ability to suppress autoimmunity by inhibiting the activation of abnormal immune cells in the gut and central nervous system (96). Tremaroli et al. demonstrated that the concentration of SCFAs, especially pentanoate, butyrate, and acetate, decreased after gastrectomy (46), which is in agreement with the findings of Meijer et al. and Nugent et al., who observed significant decreases in propionate concentrations after gastrectomy (41, 44).

#### 3.1.2 Laparoscopic sleeve gastrectomy and trimethylamine N-Oxide

TMAO is an intestinal bacterial metabolite and is important in the progression of colorectal cancer (97). Betaine, γ-butylbetaine, L-carnitine, phosphatidylcholine and choline are bioconverted to trimethylamine (TMA) by the gut microbiota. Then, TMA is oxidized in the liver to form TMAO (98). However, studies by Lee et al. (47), Gralka et al. (48), and Tremaroli et al. (46) did not reveal significant changes in these metabolites (including TMAO) in patients who underwent gastrectomy.

#### 3.1.3 Laparoscopic Sleeve Gastrectomy and Bile Acids

Bile acids play an essential role in fat metabolism. A study by Steinert et al. demonstrated varying increases in basal and postprandial plasma bile acid levels in patients undergoing bariatric surgery (gastrectomy and RYGB surgery), an increase in PYY and GLP-1 secretion, weight loss, and improvement in glucose abnormalities. Similar but more modest increases in both basal and postprandial plasma bile acid levels are also observed after gastrectomy (49). Jahansouz et al. found that bile acid levels were significantly elevated after vertical sleeve gastrectomy, with equally significant increases in unconjugated and conjugated bile acid levels (50). Khan et al. showed that adolescents lost a significant amount of weight and had increased levels of fibroblast growth factor 19 and bile acids after vertical sleeve gastrectomy (51).

#### 3.1.4 Laparoscopic sleeve gastrectomy and spermidine

Under normal conditions, spermidine levels are strictly regulated by different mechanisms. However, polyamine levels are susceptible to dysregulation under several pathological and stressful conditions, including the metabolic complications associated with obesity (85). Ocaña-Wilhelmi et al. showed that bariatric surgery can affect serum polyamine patterns and observed that cases of resolved metabolic syndrome postgastrectomy were related to specific alterations in the serum polyamine metabolome. Increased serum levels of spermidine and acetyl derivatives of spermine after surgery suggest that sleeve gastrectomy can alter circulating levels of these acetylpolyamines independently of the regression of metabolic syndrome after surgery (52).

### 3.2 Laparoscopic gastric bypass surgery and gut microbiota metabolites

#### 3.2.1 Laparoscopic gastric bypass surgery and SCFAs

Steensels et al. suggested that gut hormone levels in α-gust-/- mice may be modulated by increased expression of peptide sensors and glucose transporter proteins in the Roux limb and by increasing levels of propionate and butyrate that activate free fatty acid receptors in the hindgut in the cecum (53). Wijayatunga et al. demonstrated that RYGB surgery changes fatty acids and serum metabolites and then observed decreases in the serum levels of branched chain amino acids, acetone, butyrate, 2-aminobutyrate, 2-hydroxybutyrate, 2-methylglutarate, 3-hydroxybutyrate, and 2-oxoisocaproate, as well as increased concentrations of serum glycine, alanine, pyruvate, and taurine 6 months postsurgery in patients with morbid obesity (54). Meijer et al. also demonstrated that significant reductions in SCFA concentrations, including acetate, propionate, butyrate and valerate, occur directly following gastric bypass surgery (41). Yu et al. demonstrated that after gastric bypass surgery, the levels of butyrate or isobutyrate, caffeine and caffeine-derived metabolites (e.g., paraxanthine and theophylline), pyrraline, nicotinate ribonucleotide, fructose, indole derivatives, lysophospholipids, nucleotides and their metabolites were significantly reduced, and the levels of drug metabolites were significantly increased (42). Liou et al. showed that transplantation of fecal microbiota from RYGB mice to germ-free mice resulted in a significantly lower rate of overall obesity in RYGB recipient (germ-free) mice. Weight loss was greater in RYGB recipient mice than in recipient mice that received microbiota transplants from sham-operated animals. The researchers found higher concentrations of propionate production but lower levels of acetate production in RYGB and RYGB-R mice, and they proposed that the reduced adiposity in the RYGB-R mice may be attributed to the lower availability of acetate for lipogenesis (25).

Tremaroli et al. demonstrated that SCFAs, including propionate, butyrate and acetate, were present in certain proportions, but their levels gradually decreased after gastric bypass surgery (46). Similarly, Meijer et al. (41) and Yu et al. (42) demonstrated that propionate concentrations decreased significantly after gastrectomy. However, a study by Mukorako et al. showed that gastric bypass surgery initiated changes in the gut microflora that resulted in elevated levels of propionic, butyric, isobutyric, and isovaleric acid in the stool (55).

#### 3.2.2 Laparoscopic gastric bypass surgery and inosine

Inosine was one of the first nucleosides discovered and is also an important secondary metabolite in purine metabolism (99). The regulation of the adenosine pathways is crucial for the maintenance of immune homeostasis. First, ADP and ATP are products containing proinflammatory adenosine. Second, cAMP is a product containing anti-inflammatory adenosine. A study of alterations in one-carbon metabolism after bariatric surgery showed that the purine degradation process was limited due to the decrease in guanine deaminase and xanthine levels after gastric bypass surgery. In one study, the content of purine metabolites such as AMP, ADP, ATP, and inosine monophosphate significantly increased in rats after gastric bypass surgery (57). In another study, nucleotide metabolism profiles displayed increased serum inosine and adenosine levels after laparoscopic gastric bypass surgery, followed by decreased levels of the relevant degradation products xanthine, hypoxanthine, allantoin, and urate (56).

#### 3.2.3 Laparoscopic gastric bypass surgery and TMAO

TMAO, a metabolite of the intestinal microflora, has become a potential risk factor for cardiovascular disease and other chronic diseases. Several studies have indicated an increase in TMAO levels after gastric bypass surgery. Narath et al. demonstrated that after gastric bypass surgery, the contents of TMAO, phosphatidylcholine and indoxyl-sulfate increased, while the contents of phenylalanine, tyrosine, alanine, and branched chain amino acids choline decreased (58). Trøseid et al. reported that the levels of the microbial community-dependent metabolites TMAO and γBB increase after RYGB (59). In addition, researchers conducting a Korean obesity surgical treatment study found that RYGB surgery led to significantly higher serum levels of TMAO, while the levels of the other precursor metabolites carnitine and betaine were unchanged (47). One year after the bariatric procedure (RYGB), plasma TMAO levels were found to be significantly higher in both women and men than presurgery levels. Finally, Modesitt et al. found a 9-fold increase in TMAO levels after gastric bypass surgery (56).

#### 3.2.4 Laparoscopic gastric bypass surgery and bile acids

Gastric bypass surgery alters the flow of bile acids, enabling delayed contact with ingested nutrients and leading to severe disturbances in bile acid metabolism. For example, Kohli et al. showed a significantly higher plasma bile acid concentration in patients after RYGB (60). Broek et al. reported that elevated plasma bile acid levels and increased gut bile acid signaling to promote GLP-1 release were associated with the positive health effects of RYGB surgery (61). Yan et al. also showed that RYGB improved the metabolic profile associated with increased serum bile acid levels and hepatic FXR receptor expression in a type 2 diabetic rat model. Chávez-Talavera et al. demonstrated that RYGB increased systemic but not portal bile acid concentrations by reducing hepatic bile acid uptake in minipigs (62). In the study by Tremaroli et al., postprandial bile acid levels were slightly higher in patients after gastroplasty than in normal subjects, but the elevation was more pronounced in patients who underwent RYGB surgery (46). Pournaras et al. (63) and Patti et al. (64) both indicated that RYGB and biliopancreatic diversion increase fasting and postprandial bile acid levels by two- to threefold and that their composition also changed in parallel.

#### 3.2.5 Laparoscopic gastric bypass surgery and spermidine

A study on the impact of bariatric surgery on circulating and cardiac metabolites revealed RYGB surgery altered plasma bile acid amino acids, phosphorylcholine, glycogen, energy-related metabolites, amino acids, amine metabolites, and nucleoside metabolites (100).

### 3.3 Adjustable gastric band and gut microbiota metabolites

#### 3.3.1 Adjustable gastric band surgery and SCFAs

Butyrate production in ruminant forestomach and the large intestine is dependent on bacterial butyryl-CoA/acetate-CoA transferase activity and is greatest when there is a balance between nonstructural carbohydrates and fermentable fiber. Gastrointestinal epithelial cells apparently use butyrate and butyrate-induced endocrine signals to adapt their proliferation, differentiation, and apoptosis based on the bacterial communities (101). Studies have shown that the levels of straight-chain SCFAs, including valerate, butyrate, acetate and propionate, are significantly lower after adjustable gastric band surgery (42). Another study found that the levels of straight-chain SCFAs (such as butyrate, propionate, and acetate) decreased, and those of branched-chain SCFAs (such as isocoproate, isovalerate, and isobutyrate) increased (41).

#### 3.3.2 Adjustable gastric band surgery and bile acids

Bile acids, as an essential constituent of bile, are instrumental for the intestinal absorption of lipophilic nutrients (102). Bariatric surgery that changes the digestive tract can result in elevated primary and secondary bile acid levels in plasma. Kohli et al. analyzed alterations in bile acid levels after RYGB and laparoscopic adjustable gastric band surgery and found that plasma bile acid levels more than doubled in patients after RYGB but did not increase in patients after laparoscopic adjustable gastric bands (43). Similarly, a study by Pournaras et al. (63) on adjustable gastric banding showed no change in circulating bile acid levels in patients after adjustable gastric banding surgery. Conversely, Nakatani et al. (66) and Thöni et al. (67) suggested that elevated serum bile acid content was observed in patients undergoing gastric banding and that bile acid concentrations were positively related to postoperative GLP-1 and GIP levels. However, Kohli et al. found a trend toward lower fasting and postprandial serum bile acids in patients with severe obesity after adjustable gastric banding (60).

### 3.4 Gastric balloon surgery and gut microbiota metabolites

Gastric balloon surgery is also a common bariatric procedure, but relatively little research has investigated its impact on the gut microbiota. Currently, there are no articles reporting on alterations in the gut microbiota and its metabolites after intragastric hydrosphere surgery. To explore the deeper importance of this surgery, more studies are needed.

### 3.5 SADI-S and gut microbiota metabolites

#### 3.5.1 SADI-S and SCFAs

Bariatric surgery, particularly low-absorptive bariatric surgery, directly alters the relative abundance of gut microbes and the composition of the gut microbiota and can directly lead to changes in the levels of gut microflora metabolites, such as SCFAs. SADI-S, one of the low absorption bariatric procedures, promotes the body’s production of a profile of SCFAs (such as isobutyrate, butyrate, isovalerate, valerate and propionate) that stimulate PYY secretion and beneficial metabolic effects (55).

#### 3.5.2 SADI-S and bile acids

SADI-S can affect the metabolism of bile acids. In rats with type 2 diabetes mellitus treated with SADI-S, the levels of lithocholic acid and cholic acid were markedly improved, while the levels of taurochenodeoxycholic acid and deoxycholic acid were markedly decreased (68). The regulation of bile acid metabolism by SADI-S is beneficial for treating type 2 diabetes mellitus.

### 3.6 SIPS and gut microbiota metabolites

To date, no studies have reported changes in the gut microbiota and its metabolites after SIPS. More research is needed to explore changes in the gut microbiota and their metabolites after SIPS surgery.

## 4 Microbially modified metabolites and antitumor immunity

Obesity is an epidemic disease caused by an imbalance in energy metabolism and is highly correlated with cancer risk and the development of many tumor types. In 2017, the US Centers for Disease Control and Prevention released a report indicating that obesity-related cancers, including thyroid, kidney, liver and ovarian cancers, account for approximately 40% of cancers diagnosed in the United States (103). Furthermore, a study by Hopkins et al. revealed that obesity was strongly correlated with endometrial, breast, pancreatic, prostate and colon cancers. Obese patients have a higher risk of developing cancer, and obese patients with cancer have a higher risk of death (104). Obesity drives carcinogenic processes in the body by causing systemic metabolic dysregulation, gut microbiota disruption and immune dysfunction. Furthermore, there are closely related molecular mechanisms between obesity and cancer, including chronic inflammation, increased leptin levels, hyperinsulinemia, oxidative stress, DNA methylation, exosome miRNA release, and sexual hormone metabolism (105). Importantly, adipose tissue can act as a chemokine that can regulate inflammation, tumor behavior and the tumor microenvironment. Moreover, adipose tissue stimulates the body to secrete a variety of hormones, including lipocalin and leptin (106). Therefore, reducing obesity is important for cancer prevention. A study by researchers from Harvard Medical School and collaborators suggested that a high-fat diet reduced the number of CD8+ T cells and their antitumor activity in the tumor microenvironment (107). This is because cancer cells adapt to increased fat levels by redesigning their metabolism to better obtain energy-rich fat molecules from T cells, inhibiting T-cell metabolism and accelerating tumor growth.

The efficacy of immunotherapy is closely related to the tumor microenvironment. The tumor microenvironment is a complicated integrated system comprised of tumor cells, tumor-associated fibroblasts, and cytokines (108) and contains various types of immune cells, including T cells, NK cells, macrophages, and dendritic cells (109). However, the tumor microenvironment can be altered by tumor cells to enable escape from the immune system. Indeed, tumor cells can escape the monitoring, recognition, and attack response of the immune system by changing the tumor microenvironment to favor tumor growth (110). The malignant biological behaviors of tumors are accelerated once they bypass immune surveillance, leading to increased tumor metastasis, invasion, and proliferation. Immunosuppression is a major obstacle to antitumor immunotherapy.

Microbially modified metabolites are components of an important pathway through which the gut microbiome affects tumor immunity. Recent studies have suggested that microbially modified metabolites participate in tumor immunity and have a great influence on the efficacy of tumor immunotherapy (**Table 2**). In obese patients, gut microbiota-modified metabolites have the potential to promote energy absorption and metabolism. These metabolites enter the bloodstream through the intestines and flow through the body in the circulating blood, directly or indirectly affecting the body’s metabolism and immune processes (39). Microbially modified metabolites might reduce tumor immunosuppression by regulating metabolism in the tumor microenvironment, and more precise regulation of tumor immunity could potentially be achieved by regulating these gut-derived compounds. There is widespread interest in how gut microbiota-modified metabolites directly influence body weight and participate in metabolic and immune activities.

**Table 2:** Studies on microbially modified metabolites for promoting tumor immunotherapy.

Beneficial Metabolites	Targets	Tumor Types	Tumor Immunotherapy	Reference
Butyrate	CD8^+^ T-cell	Mc38 colon tumor	Anti-PD-L1	(111)
Pentanoate and butyrate	CD8^+^ CTL and CAR-T cells	Melanoma and pancreatic tumors	CTL and CAR-T	(112)
TMAO	tumor cells and CD8^+^ T-cell TAM and effector T cells	TNBC PDAC	Anti-PD1 Anti-PD-1 and/or anti-Tim3	(113) (114)
Primary bile acids	NKT cell	Hepatocellular tumor	–	(115)
Gallic acid	Treg cells	Colorectal tumor	Anti-PD-1	(116)
Exopolysaccharide	CCR6+ CD8+ T cells	CCL20-expressing tumors (Colon26 and 4T1 tumors)	Anti-CTLA-4 or anti-PD-1	(117)
Spermidine	CD8^+^ T-cell	MC38 tumor	Anti-PD-L1	(118)

### 4.1 SCFAs

The production of high SCFA levels in the gut leads to higher insulin sensitivity, glucose tolerance and energy expenditure in diabetic and obese animals, improving metabolism, reducing obesity, further enhancing immunity and exerting anticancer effects (119–121). The main effects of SCFAs on host health include 1. preventing microbial invasion and reducing susceptibility to infection by maintaining a structurally sound and intact mucus layer (122); 2. reducing chronic inflammatory disease and maintaining a functional immune system by decreasing luminal oxygen levels; and 3. stimulating the production of colonic regulatory T cells (Tregs) in a GPR41- and GPR43-dependent manner, which modulates the thymic microenvironment and induces the expression of autoimmune regulators, directly affecting the immune system (123).

SCFAs, such as butyrate, are produced by the decomposition of polysaccharides and dietary fiber in the colon. Butyrate can induce apoptosis in lymphoma cells and colorectal cancer cells (124, 125). CD8+ T cells are critical in tumor immunity. Therefore, it is particularly important to study how to improve the amount and function of CD8+ T cells during the treatment of tumors. Butyrate was found to enhance the chemotherapy benefits of oxaliplatin by strengthening the immune response of CD8+ T cells (111). Butyrate can increase ID2 expression in CD8+ T cells. Further mechanistic study demonstrated that ID2 elevates the function and proliferation of CD8+ T cells by regulating the IL-12 signaling pathway. Butyrate treatment can also promote the therapeutic effect of oxaliplatin in AOM-DSS mice and the PD-L1 antibody in Mc38 colon cancer mice. Luu et al. showed that the SCFAs butyrate and pentanoate enhanced adoptive immunotherapy with chimeric antigen receptor (CAR) and CD8+ cytotoxic T lymphocyte (CTL) T cells (112). The CTLs and CAR-T cells treated with butyrate and pentanoate exhibit enhanced activation of mTOR signaling and inhibited activity of class I histone deacetylase. Epigenetic reprogramming and metabolism improve the production of effector molecules such as TNF-α, CD25 and IFN-γ. Therefore, pentanoate and butyrate enhanced the antitumor efficacy of CAR-T cells and CTLs in pancreatic cancer and murine melanoma models.

### 4.2 Inosine

Intestinal *B*. *pseudolongum* has the ability to increase its response to immunotherapy by producing the metabolite inosine (126). Inosine promotes immune checkpoint blockade (ICB) therapy by affecting the adenosine A2A receptor on T cells in addition to the required costimulation, promoting the effect of anti-CTLA4 in reducing tumor size and increasing IFN-γ+CD8+ and IFN-γ+CD4+ T-cell infiltration in mouse models of Msh2^LoxP/LoxP^Villin-Cre tumors, MB49 bladder tumors, and heterotopic melanoma. Furthermore, inosine acts as an alternative carbon source to support the growth of efferent T cells in glucose-deficient media (127). Inosine improves antitumor activity mediated by efferent T cells in animal models, providing potential strategies to optimize immunotherapy through metabolic regulation. In addition, inosine enhances tumor cell immunogenicity by inhibiting the activity of the ubiquitin activating enzyme UBA6, thereby improving the tumor immunotherapy response (128). Inosine supplementation could improve the sensitivity of murine melanoma and breast tumors to ICB, providing a strategy to overcome ICB resistance. Moreover, inosine is useful for weight loss, as it is observed to induce the browning of white adipose tissue (91).

### 4.3 TMAO

TMAO is considered a harmful microbial metabolite that increases the risk of inflammation-related diseases such as cardiovascular disease. Triple-negative breast cancer (TNBC) is an aggressive disease with a poor prognosis, and immunotherapy has achieved limited success in this group of patients. TMAO concentration was observed to be higher in tumors with an activated immune microenvironment. Furthermore, higher plasma TMAO concentrations resulted in better responses to immunotherapy in patients (113). Specifically, TMAO induces tumor cell pyroptosis by activating the endoplasmic reticulum stress kinase PERK, thereby enhancing CD8+ T-cell-mediated antitumor immunity in TNBC *in vivo* (113).

Pancreatic ductal adenocarcinoma (PDAC) is associated with a high fatality rate. Immunotherapy cannot ameliorate clinical outcomes in PDAC patients due to the highly immunosuppressive tumor microenvironment. However, TMAO may enhance the response to ICB and antitumor immunity in cases of PDAC (114). In one study, TMAO induced an immunostimulatory tumor-associated macrophage (TAM) phenotype and activated the function of effector T cells in the tumor microenvironment in a type I interferon-dependent manner.

### 4.4 Bile acids

Bile acids are key ligands involved in the regulation of TGR5 and FXR during glucose and lipid metabolism, influencing several different metabolic processes in the body and effectively improving obesity, insulin resistance and fasting glucose levels (129, 130). Similarly, bile acids are a critical component of gut microbiota metabolites involved in improving the host’s immune system. Bile acid receptors are expressed in a variety of innate immune cells, and they are involved in fine tuning the responsiveness of these cells to bacterial and endogenous antigens (131). Kawamata et al. have shown that bile acid-activated receptors can affect monocyte and macrophage effector functions by regulating the bile acid receptors GPBAR1 and FXR (132). Furthermore, Vavassori et al. showed that bile acids can activate FXR, reduce the differentiation of dendritic cells in the intestine and rescue mice from gut inflammation (133). Mencarelli et al. demonstrated that bile acids activated the functional expression of FXR in hepatic NKT cells, severely inhibiting the production of a proinflammatory mediator (osteopontin) and thus reducing inflammation (134). Furthermore, several studies have suggested that the bile acid receptors GPBAR1 and FXR are negative regulators of NF-kB and NLPR3 inflammatory vesicles (135, 136). Bile acids also play an immunomodulatory role by influencing T-cell differentiation and activity in the gut. Hang et al. illustrated that bile acid metabolites could activate TH17 cells and Treg cells, which each regulate the immune response by suppressing or promoting inflammation (137). Another study revealed that bile acid metabolites could increase the number of FOXP3+ Treg cells to inhibit intestinal inflammation (138). Ultimately, bile acids play the same immune-enhancing role.

Bile acids are abundant in the intestinal tract of mammals and can be converted by intestinal bacteria into a large number of small molecules with biological activity. Hang et al. screened bile acid metabolite compounds (137) and found that two different metabolites of lithocholic acid (LCA), isoalloLCA and 3-oxoLCA, regulate T-cell functions in mice. Furthermore, primary bile acids are observed to increase CXCL16 expression, inducing the aggregation of NKT cells in the liver (115). Therefore, liver cancer was effectively inhibited. However, secondary bile acids produced by intestinal flora have the opposite effect. Therefore, tumor immunity can be enhanced by regulating bile acid metabolism.

### 4.5 Gallic acid

Regulatory T (Treg) cells mainly mediate the abilities of tumors to evade the immune response. High levels of Treg cells can suppress the immune response and accelerate the growth of tumors. Therefore, Treg cells are a potential target to promote antitumor immunity. The gut microbial metabolite gallic acid inhibits tumor growth and improves anti-PD-1 blockade effects in colorectal cancer (116). The mechanisms were elucidated as follows: gallic acid suppresses Foxp3 protein expression by targeting Usp21 in Treg cells, which decreases the levels of Th-1-like Treg cells, weakening their immunosuppressive function. Indeed, gallic acid is observed to suppress the development of tumors in AOM-DSS mice and Mc38 colon cancer mice. Moreover, gallic acid can downregulate PD-L1 protein expression in Treg cells, thereby enhancing the antitumor immunity effect of anti-PD-1 ICB.

### 4.6 Exopolysaccharide

The exopolysaccharide produced by dietary Lactobacillus is a kind of natural macromolecular compound with strong physiological activity. Exopolysaccharide shows concentration-dependent antitumor activity in a variety of tumor cells (139, 140). Its antitumor mechanism is mainly realized by strengthening immune regulation, including innate and adaptive immunity (141). The secretion of interleukin, tumor necrosis factor, interferon and other cytokines that can improve the body’s immune response is accelerated by the activation of immune effector cells (such as macrophages, lymphocytes, NK cells and dendritic cells, 142, 143). Exopolysaccharide can enhance ICB therapy by inducing the production of CCR6+ CD8+ T cells (117). IFN-γ is produced to maintain the functions of T cells. Therefore, the tumor microenvironment could enhance the antitumor effects of anti-PD-1 or anti-CTLA-4 monoclonal antibodies against CCL20-expressing tumors in mice.

### 4.7 Spermidine

Spermidine is associated with many physiological processes in cells and has immunomodulatory functions in many diseases. Yang et al. demonstrated that spermidine specifically inhibited the expression of CD80, interleukin-12 (IL-12) and interleukin-1β (IL-1β) and strengthened macrophage arginase 1 expression to alleviate experimental autoimmune encephalomyelitis (144). Additionally, spermidine can significantly inhibit the secretion of proinflammatory mediators (prostaglandin E2 and nitric oxide) and cytokines (tumor necrosis factor-α (TNF-α) and IL-1β) in lipopolysaccharide-stimulated macrophages (145). Researchers found that restoring spermidine levels boosted the immune response of B cells. Exogenous supplementation with arginine could reverse age-dependent eIF5A-TFEB-autophagy axis decline and restore B-cell functions to enhance the immune response (146).

The ability of the immune system declines with age. PD-1/PD-L1 immunotherapy is generally less effective in older adults than in younger adults. Production of the polyamine spermidine gradually decreases with age (86). Supplementation with spermidine may improve age-related diseases, including those associated with the immune system (147). In addition, spermidine supplementation may provide a way to treat obesity by regulating the gut microbiota (148). The antiaging characteristics of spermidine are attributed to its ability to regulate autophagy by reversing B-cell senescence (146). Recently, the relationship between spermidine and age-induced T-cell immunosuppression was revealed (118). Spermidine supplementation increases the antitumor activity of PD-1 blocking immunotherapy in aged mice. Spermidine was found to enhance the activity of fatty acid oxidation by directly binding and activating the mitochondrial trifunctional protein, thereby strengthening the activity of CD8+ T cells, which induced favorable antitumor activity of PD-1 blocking immunotherapy.

## 5 Future perspectives

In recent years, many studies have suggested that the gut microbiome is linked to cancer. Increasing evidence indicates that the gut microbiome is also an essential factor affecting the efficacy of tumor immunotherapy. Favorable gut microbial composition can enhance tumor immunotherapy by influencing the immune system. However, what is considered a beneficial gut microbiome varies across individuals due to geographical differences. Therefore, accurate regulation of the gut microbiome is challenging. Bacterial metabolite therapy has more specific targets and advantages than bacterial therapy. A large amount of evidence shows that obesity is strongly associated with the development and occurrence of tumors, and the success of cancer treatment in patients with obesity is limited. It is unclear what dosage should be used for patients with obesity with cancers. Moreover, the toxic side effects are unknown. Bariatric surgery is beneficial for regulating a disordered gut microbiota and metabolites. As described in this review, five types of bariatric surgery (laparoscopic sleeve gastrectomy, laparoscopic gastric bypass surgery, adjustable gastric band surgery, single-anastomosis duodenoileal bypass with sleeve gastrectomy surgery and stomach intestinal pylorus sparing surgery) can induce changes in microbially modified metabolites, such as SCFAs, TMAO, bile acids, spermidine, and inosine. Some of these changes may help explain the high success rate of bariatric surgery. Some microbially modified metabolites, such as SCFAs, bile acids, spermidine, and inosine, contribute to weight loss. However, significant weight loss may affect nutrient absorption in cancer patients, thus influencing their prognosis. Therefore, for cancer patients with obesity, the regulation of bacterial metabolites in place of weight loss surgery will be more beneficial during cancer treatment. We summarized several microbially modified metabolites that are beneficial to cancer immunotherapy. We found that SCFAs, TMAO, spermidine, inosine and bile acids were closely related to bariatric surgery and tumor immunotherapy. It is suggested that supplementation with butyrate, pentanoate, spermidine, inosine and bile acids can improve the antitumor immunity of patients with obesity and be beneficial to weight loss. Although TMAO has been shown to strengthen the effects of tumor immunotherapy, high levels of TMAO have adverse effects on human health. The effect of other metabolites, such as gallic acid and exopolysaccharide, requires further investigation.

## 6 Conclusion

The gut microbiome is closely linked to human health. Abnormal microbiomes can promote the progression of many diseases, such as cancer and obesity. Microbially modified metabolites are an important mechanism for explaining the effect of the gut microbiome on human health. In this paper, we discuss changes in microbial metabolites induced by bariatric surgery and beneficial microbial metabolites for weight loss and tumor immunotherapy. An examination of the literature revealed that supplementation with butyrate, pentanoate, spermidine, inosine and bile acids may enhance tumor immunotherapy in patients with obesity. The present study provides a strategy for simulating bariatric surgery with microbiota metabolites to achieve better tumor immunity in cancer treatment.

## Author contributions

LL, WS, and YZ conceived and designed the research. YH and MW drafted the original manuscript. MX, TL, XW, and YF revised and improved the manuscript. SL, TC, XX, and LD helped with the table and figure preparation in this manuscript. JL, CW, ZX, and XS collated the references. All authors contributed to the article and approved the submitted version.
